# Development of ELISA and Lateral Flow Immunoassays for Ochratoxins (OTA and OTB) Detection Based on Monoclonal Antibody

**DOI:** 10.3389/fcimb.2020.00080

**Published:** 2020-03-06

**Authors:** Mohamed Hassan Fadlalla, Sumei Ling, Rongzhi Wang, Xiulan Li, Jun Yuan, Shiwei Xiao, Ke Wang, Shuqin Tang, Hoyda Elsir, Shihua Wang

**Affiliations:** ^1^Fujian Key Laboratory of Pathogenic Fungi and Mycotoxins, Fujian Agriculture and Forestry University, Fuzhou, China; ^2^School of Life Sciences, Fujian Agriculture and Forestry University, Fuzhou, China; ^3^Key Laboratory of Biopesticide and Chemical Biology of the Education Ministry, Fujian Agriculture and Forestry University, Fuzhou, China

**Keywords:** ochratoxins, monoclonal antibody, hybridoma technology, ic-ELISA, colloidal gold strip, nanoflowers gold strip

## Abstract

Ochratoxins were important secondary metabolites secreted by fungi, and OTA and OTB are mainly significant mycotoxin, having toxic effects on humans and animals. Therefore, it is important to establish a rapid, sensitive, and precise method for ochratoxins detection and quantification in real samples. In this study, a stable monoclonal antibody (mAb) that recognizing both OTA and OTB toxins was employed for the establishment of indirect competitive ELISA (ic-ELISA), colloidal gold nanoparticles (CGNs), and nanoflowers gold strips (AuNFs) for detection of ochratoxins in real samples. A 6E5 hybridoma cell line stable secreting mAb against both OTA and OTB toxins was obtained by fusion of splenocytes with myeloma SP2/0 cells. The 6E5 mAb had a high affinity (3.7 × 10^8^ L/mol) to OTA, and also showed similar binding activity to OTB. The optimized ic-ELISA resulted in a linear range of 0.06–0.6 ng/mL for ochratoxins (OTA and OTB) detection. The IC50 was 0.2 ng/mL and the limit of detection (LOD) was 0.03 ng/mL. The mean recovery rate from the spiked samples was 89.315 ± 2.257%, with a coefficient variation of 2.182%. The result from lateral flow immunoassays indicated that the LOD of CGNs and AuNFs were 5 and 1 μg/mL, respectively. All these results indicated that the developed ic-ELISA, CGNs, and AuNFs in this study could be used for the analysis of the residual of ochratoxins (OTA and OTB) in food and agricultural products.

## Introduction

**Figure 1:**
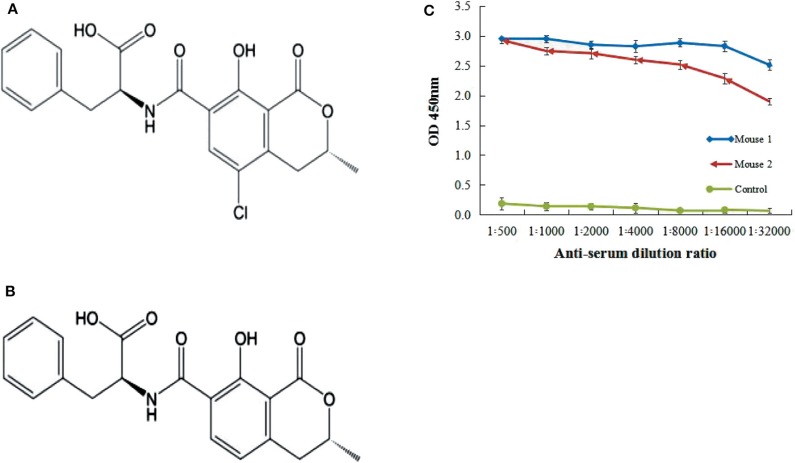
Ochratoxins structure and titer of mice serum. **(A)** Chemical structure of Ochratoxin A (OTA). **(B)** Chemical structure of Ochratoxin B (OTB). **(C)** Anti-OTA titer of serum was assayed by iELISA. Mice 1 showed the highest antibody titer as compared to control mouse.

Mycotoxins are secondary toxic substances produced by fungi in the later process of growth from different types of food, and pose great danger to human's health, animals, and crops. Serious diseases such as cancer, tumors, and general weaknesses are often arises when these toxins contaminated foods were consumpted. About 300–400 different types of mycotoxins have been identified so far, and the most famous are Aflatoxin, Ochratoxin, Patulin, Zearalenone, and Trichothecenes among them (Berthiller et al., 2007; Malir et al., 2013). Ochratoxins are a group of mycotoxins produced mainly by *Aspergillus* species and some *Penicillium* species (Heussner and Bingle, 2015). Ochratoxin A (OTA) (Figure 1A) is the most prominent family member, and the contamination with OTA mold may be directly linked to meat and dairy products (Kuiper-Goodman and Scott, 1989). Ochratoxin B (OTB) is a non-chlorinated form of ochratoxin A (OTA) (Figure 1B) (Heussner and Bingle, 2015). The risk lies in the indirect contamination of meat and meat products by animals exposed to OTA-infected feed. This is mainly related to chickens, pigs, and small ruminants that have not fully developed their gastrointestinal flora.

The widespread occurrence and noted toxicity of ochratoxins have prompted researchers to develop rigorous analytical methods for its control. The most frequent, reproducible, and accurate methods applied for detecting ochratoxins residue in real samples are thin layer chromatography (TLC), high performance liquid chromatography (HPLC), and gas chromatography (GC), paired to ultraviolet/visible, mass spectrometry (MS) or fluorescence detection (Soleas et al., 2001; Turner et al., 2009; Barthelmebs et al., 2011; Luan et al., 2016). Instrument-based methods present good sensitivity, selectivity and can be used in simultaneous analysis of multiples toxins. However, these methods exhibited complex equipment, incompatibility with real samples, cost for analysis, and amount of time required. Recently, new technologies coupled with immunochemical assays have been proposed for rapid and sensitive monitoring and quantifying of OTA analysis in contaminated food and beverages (van der Gaag et al., 2003), including enzyme immunoassays (de Saeger et al., 2002; Radoi et al., 2009), fluorescence polarization immunoassays (Shim et al., 2004; Zezza et al., 2009), immunosensors (Alarcón et al., 2006; Ricci et al., 2007; Prieto-Simón et al., 2008; Radi et al., 2009), and aptamer-based assays (Rivas et al., 2015).

In recent years, detection methods based on monoclonal antibody (mAb) with the advent of hybridoma technology, have been used for detection of mycotoxins, because of their adaptability, rapidity, simplicity, specificity, sensitivity, low cost, and compatibility with real samples (Lupo et al., 2010; Le et al., 2012). Immunoassay with colloidal gold nanoparticles (CGNs) is visible, rapid, and easily operated, because CGNs could be detected by the naked-eyes and are easily prepared. Consequently, immunoassay method based on colored colloidal gold-antibody conjugates is one of the most widely methods used in mycotoxins detection (Luan et al., 2015; Liu et al., 2018; Pei et al., 2018), and this immunochromatographic assays would be completed by one step within 5 min without complex procedures, which can meet on-site testing requirements. Multi-branched gold nanoflower (AuNFs) with large specific surface area was also applied in immunoassay to improve the sensitivity of detection (Dondapati et al., 2010; Guerrero-Martínez et al., 2011; Ji et al., 2015; Pei et al., 2018). Hence, the main aim of this study was to produce a monoclonal antibody against ochratoxins (OTA and OTB) with high affinity, and to establish sensitive ic-ELISA, CGNs, and AuNFs immunoassays for detection of ochratoxins (OTA and OTB) in real samples.

## Materials and Methods

### Chemicals and Reagents

Ochratoxin A, Ochratoxin B, Ochratoxin A-BSA conjugates, Keyhole limpet haemocyanin (KLH), Bovine serum albumin (BSA), Ovalbumin (OVA), Chloroauric acid (HAuCl_4_∙4H_2_O), Goat anti-mouse-peroxidase conjugate (IgG-HRP), mouse monoclonal antibody isotyping kit (IgG1, IgG2a, IgG3, IgM, IgA), fetal bovine serum (FBS), and RPMI 1640 were purchased from Sigma-Aldrich Chemical (St. Louis, MO, USA), while OTA-KLH and OTB-KLH were purchased from Sinopharm Chemical Reagent (China). Myeloma cell line SP2/0 was obtained from our laboratory. All other reagents were chemical grade and obtained from commercial sources in china.

### Experimental Animals, Immunization and Serum Collection

Female *Balb/*c mice (5-week-old) were purchased from Wushi animal laboratory (Shanghai, China). A total of six mice, divided into two groups, were immunized after they are 7-week age. The OTA-BSA conjugates (100 μg) were emulsified with an equivalent volume of Freund's complete adjuvant immediately prior to injection. The mixer used for the first injection was administered at multiple sites intraperitoneally (i.p.). Then, another five to boost immunization (subcutaneously) with Freund's incomplete adjuvant were carried out in equal volumes at 2 weeks intervals. The mice were bled with minimized risk and serum of each immunized mouse was collected and centrifuged at 4,000 r/min for 30 min. The supernatant was stored at −20°C until required. All the animal studies were conferring to the Committee for Animal Ethics of Fujian Agriculture and Forestry University (FAFU) in China (C1017/23.12.2014).

### Indirect ELISA for Detection of Serum Titer

In this indirect ELISA method, the OTA-KLH (1 μg/mL) was dissolved in 0.05 M Na_2_CO_3_, pH 9.6, and 100 μL/well this solution was added into micro-titer plate at 37° for 2 h or at 4°C overnight. After decanting the antigen solution, the plate was washed 3 times with washing buffer. Non-well binding sites were blocked with blocking buffer, and then incubated as described above. The solution was discarded and washed 3 times with PBS-Tween and another three times with PBS. Then, 100 μL/well of test and control serum with serial dilution was added into the plate for 2 h. After washing plate, HRP-goat anti-mouse IgG was added into the plate (1:8,000, 100 μL/well) and incubated at 37° for 2 h. Then the plate was washed, and newly TMB was prepared and added (100 μL/well). After 15 min incubation at 37° in the dark, stop solution (2 M H_2_SO_4_, 50 μL/well) was added, and then optical density was examined at 450 nm on a Micro-plate Reading machine (Jin et al., 2014).

### Cell Fusion and Screening of Positive Hybridoma Cell

The hybridoma cell against ochratoxins was screened according to the standard method (Jin et al., 2014; Ling et al., 2014) with slight modifications. After five times boosting, the mouse with the highest titer was selected and administered i.p. with OTA-BSA without adjuvant 3 days prior to the cell fusion. Freshly isolated B-lymphocytes from the immunized mouse were isolated and fused with Sp2/0 myeloma cells at 1:10 ratio using 1 mL 50% polyethylene glycol (PEG-1450) (Cho et al., 2005). Hybridoma cells were thoroughly cultured into 96-well plates in the presence of feeder cells. Positive cells were sub-cloned 3 times and isolated from the culture by limited dilution method. The positive hybridoma was cloned for a second time and then expanded (Ling et al., 2014).

### Isotyping and Chromosome Analysis of the Positive mAb

After subclone of the desired hybridoma cells, the isotyping analysis of positive hybridoma was carried out according to the method previously described (Ling et al., 2014). Chromosome was analyzed using Giemsa staining (Kozak et al., 1977) for chromosome count of positive hybridoma, and inverted microscope was used for chromosome number determination (Kozak et al., 1977; Ling et al., 2014).

### Purification of mAb From Ascites

For generation ascites containing mAb, adult female Balb/c mice were primed intraperitoneally with 0.5 mL pristine. After 7 d, the primed mice were received i.p. 6E5 hybridoma cells. About 1 week later, the developed ascites fluid was carefully harvested and centrifuged at 10,000 r/min for 30 min. The ascites were purified with two-step caprylic/ammonium sulfate precipitation methods (Liu et al., 2013). The purity of the mAb was assayed by SDS-PAGE (Chang and Gottlieb, 1988; Di Girolamo et al., 2010), and stained using Coomassie Brilliant Blue staining solution. Protein concentration of purified mAb was assayed via BCA kit (Jin et al., 2014; Ling et al., 2014).

### Affinity and Cross-Reactivity of the Positive mAb

An affinity test for mAb was assessed according to the previous publications (Jin et al., 2014; Ling et al., 2014) with minor modifications. Four different concentrations (5, 2.5, 1.25, and 0.625 μg/mL) of the OTA-KLH was coated in ELISA plate, and mAb was sequentially diluted with PBSM and added to the reaction well. After washing, the HRP conjugated goat anti-mouse IgG (1:8,000 dilutions) was added, and the residual steps were the same as above. The affinity constant of this mAb was evaluated by a method reported previously (Beatty et al., 1987). The specificity and cross-reactivity of this mAb were carried out according to previous method (Ling et al., 2014, 2015). Several mycotoxins other than OTA and OTB, such as Zearalenone (ZEN), Deoxynivalenol (DON), Fumonsins B1 (FB1), and fusarochromanone (FC) were used as competitor antigens for 6E5 mAb, and cross-reactivity was calculated as: cross-reactivity (%) = concentration of standard OTA inhibiting 50% of antibody binding divided by the concentration of competitor inhibiting 50% of antibody binding multiplied by 100% (Cho et al., 2005).

### Standard Curve and Real Samples Detection by ic-ELISA

To further test the sensitivity of the developed ic-ELISA method, standard OTA or OTB toxin and the mAb were mixed together and dropped into ELISA plates. By using OriginPro 9.1 (OriginLab, Northampton, MA, USA), the standard curve was constructed. The concentration of OTA toward inhibition (20–80%) was used as working range for detection (Kido et al., 2008). Matrix effect in standard curve was also tested in the study. Methanol was chosen for extraction by the developed ic-ELISA. Matrix effect was minimized by diluting the samples before the ELISA assay. In the study, Corn samples without OTA contamination tested by GC–MS (data not show) was analyzed by testing 10- to 500-fold dilutions. The samples were diluted to minimize the matrix effect in ELISA assay, and this reduced matrix effect was compared with the prepared standard curve (in PBS buffer) (Ling et al., 2014). The efficiency of standard curve was used for recovery test. Real corn samples were randomly selected from local market, 1 g of the corn powder samples with non-detectable OTA by GC–MS were artificially spiked with OTA at different concentrations, and maintained at 4 _C overnight. Then, samples were mixed with 5 mL methanol-water (7: 3, v: v) and incubated at room temperature for 30 min. After that, the suspension was obtained by centrifuging the mixture at 8,000 r/min for 20 min and the corn extracts were determined by ic-ELISA (Jin et al., 2014). The recovery rate was made according to the standard curve. Corn and related samples were collected randomly, and the corn extracts were determined by ic-ELISA.

### Construction and Identification of Colloidal Gold Strip Test (CGNs)

To synthesize the mAb-colloidal gold conjugates, 40 nm well-dispersed CGNs were prepared in this study and more details on the preparation of CGNs were described in Supplementary 1. CGNs has four components, including nitrocellulose (NC) membrane, sample, absorbent, and conjugate pads (Wang et al., 2014). The Millipore 135 NC membrane was used to coat the OTA-BSA conjugate (as a test line) and HRP-conjugate (as a control line). The conjugate pad was used to apply gold colloid-antibody conjugates at rate of proper spray. To evaluate the cross-reactivity of the test strips, the related mycotoxins of OTB, CTN, FB1, DON, and ZEN were used to interact with the mAb-colloidal gold which was sprayed into conjugate pad according to the different mycotoxins, respectively. After 10 min incubation at room temperature, the reaction was carried out and the results were detected by naked eye. For sensitivity test (Ling et al., 2015), 10, 5, 2.5, 1.25, and 0 μg/mL concentrations of OTA were allowed to react with the colloidal gold-antibody conjugates. The LOD for OTA was determined using CGNs method. To assay the OTA in real samples, six corn samples purchased from supermarket were detected by CGNs according to the previous methods with some modifications (Ling et al., 2014).

### Construction and Characterization of Nanoflowers Gold Strip Test

AuNFs was prepared according to procedure previously described with some modifications (Perrault and Chan, 2009) and characterized to show its shape by transmission electron microscopy (TEM) as described in Supplementary 1. Firstly, gold seeds was prepared according to the same procedure described before (Wang et al., 2014). For labeling AuNFs, the mAb was applied to conjugate with AuNFs (Yokota et al., 1992). The pH of AuNFs solution was adjusted to 9.0 with 0.2 mol/L K_2_CO_3_, and then mAb was added dropwise to 10 mL AuNFs solution under gentle stirring for 30 min. AuNFs also contained four parts, such as nitrocellulose (NC) membrane, sample, absorbent, and conjugate pads. Conjugate of mAb-AuNFs were sprayed into pads, and OTA-BSA conjugate and HRP- conjugate were immobilized on the NC membrane as the control and test lines, respectively. Several mycotoxins other than OTA and OTB, such as CTN, FB1, DON, and ZEN were used to determine the cross-reactivity of the strip assay. The different concentrations of OTA were used for the sensitivity and LOD test (Ling et al., 2015). For examining the robustness of AuNFs gold strip, six corn samples were used for this detection.

## Results and Discussion

### Mice Immunization and Titer Determination

To provoke the immune system of female Balb/C mice, an immunogen of OTA-BSA conjugate was used to inject mice. The titer of anti-OTA-BSA serum isolated from the immunized mice was assayed by indirect (non-competitive) ELISA (iELISA) using OTA-KLH as coating antigen. The collected serum showed the highest titer response to OTA toxin compared to non-injected (negative control) mice (Figure 1C), indicating that the OTA-BSA conjugates had successfully elicited an adequate immune response. Therefore, this study was performed with OTA-BSA as immune antigen while OTA-KLH as coating antigen.

### Cell Fusion and Screening of Hybridoma Cells

The mouse with the highest anti-serum titer was sacrificed for splenocytes isolation, and hybridoma cells were generated by PEG-mediated cell fusion. The supernatants taken from growing hybridoma cells were assessed for specific antibody production against plates coated with OTA-KLH, and the positive hybridomas were screened by indirect ELISA. Seventeen clones gave positive ELISA values, and these were expanded and further screened against OTA-KLH. Three hybridoma cells: 6E5, 10D3, and 8F5 with higher titer were sub-cloned by limiting dilution, and screened by indirect ELISA. Finally, the hybridoma cell line 6E5, which stably secreted monoclonal antibody, was chosen for further research. A scheme of the results was outlined in Supplementary 1.

### Isotypes and Chromosome Analysis

**Figure 2:**
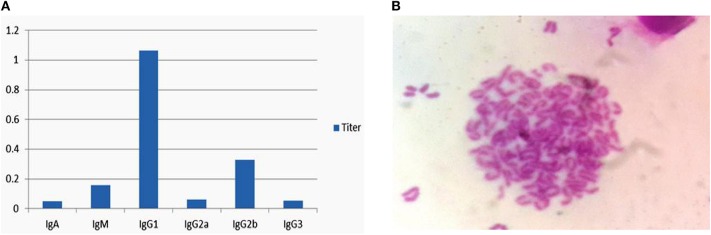
Isotypes and chromosome analysis of hybridoma cell 6E5. **(A)** Isotyping of 6E5 cell by using an isotyping kit. **(B)** Chromosome analysis of hybridoma cell 6E5.

The above hybridoma cells (6E5) were used for antibody subclasses analysis using a commercial isotyping kit (IgG l, IgG2a, IgG2b, IgG3, IgA, and IgM), and this 6E5 was found to secrete antibody of the IgG1 subclass as shown in Figure 2A. The chromosome count analysis in Giemsa dye showed that the chromosome numbers of the hybridoma cell 6E5 were 102 ± 4 (Figure 2B). Among the experimental replicates, this hybricoma cell was produced by fusing Sp2/0 myeloma cells (chromosome number 39 ± 1) with spleen cells (chromosome number 66 ± 4) (Zhou et al., 2009). Therefore, the result of the chromosome number showed that the positive clone 6E5 was a hybridoma cell produced from the fusion of Sp2/0 myeloma and spleen cell.

### Purification of Positive mAb

**Figure 3:**
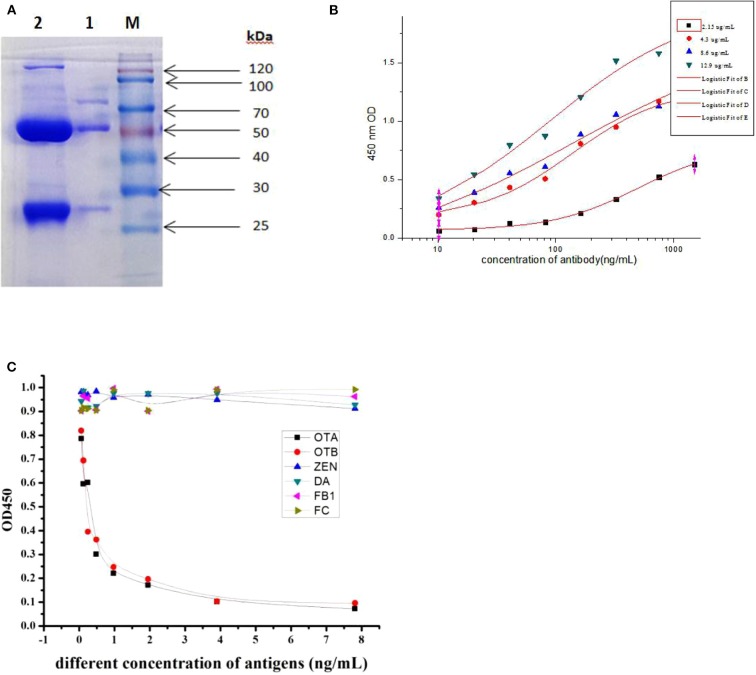
Cross-reactivity and affinity of mAb from 6E5. **(A)** Purification analysis of mAb from ascites by sodium dodecyl sulfate-polyacrylamide gel electrophoresis (SDS-PAGE). Lane M, standard protein marker. Lane 1, purified mAb. Lane 2, unpurified ascites fluid. **(B)** Affinity result of mAb at different concentration of coating antigen. **(C)** Cross-reactivity of purified mAb to structurally related mycotoxin.

The hybridoma clone 6E5 was grown as ascites by intraperitoneal (i.p.) injection into the initially primed mice. The ascitic fluid containing monoclonal antibodies were withdrawn aseptically, and the 6E5 mAb was purified by two-step octanoic acid/ammonium sulfate methods. The purity of the mAb was determined by sodium dodecyl sulfate-polyacrylamide gel electrophoresis (SDS-PAGE) as in Figure 3A, showing the heavy and light chain bands at 50 and 27 kDa, respectively. Subsequently iELISA was used to determine the activity of this mAb from the crude and purified 6E5 mAb. The results indicated that the 6E5 mAb had high activity and successfully captured OTA antigen coated in ELISA plate. Furthermore, the purified mAb presented high titer (above 3 × 10^4^), and the concentration of the purified 6E5 mAb was determined to be 2.25 mg/mL. All these results indicated that the positive mAb was successfully purified at a high level.

### Cross-Reactivity and Affinity of Positive mAb

The affinity of the 6E5 mAb against OTA was characterized based on iELISA assay at different concentrations of the OTA antigen (2.15, 4.3, 8.6, and 12.9 μg/mL) when OTA-KLH was used as coating-antigen. Analysis of the affinity constant of 6E5 mAb for OTA was obtained using the Microcal Originpro software version 9.1. The evaluated affinity results revealed that this mAb secreted by 6E5 hybridoma clone was sensitive to OTA, and the affinity constant of 6E5 mAb was 3.7 × 10^8^ L/mol (Figure 3B). To measure the cross-reactivity of the 6E5 mAb, competitive inhibition ELISA was performed toward structurally related mycotoxins which sharing common epitopes (antigens). The results from Figure 3C showed that this mAb was capable of binding Ochratoxin A (OTA) with almost cross-reactivity of 98% to OTB, as the OTA and OTB have similar structure and common antigen epitope, whereas there was no cross-reactivity with other mycotoxins including Zearalenone (ZEN), Citrinin (CTN), Deoxynivalenol (DON), and Fumonsins B1 (FB1). The above result further indicating that the prepared mAb was specific to both OTA and OTB, and this mAb can be used to detect ochratoxins contamination in real samples.

### Preparation of Standard Curve

**Figure 4:**
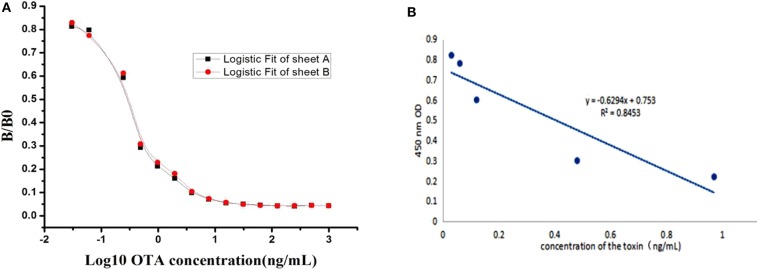
Standard curves for OTA detection. **(A)** A typical calibration curve illustrated by plotting (B/B0) against OTA concentration. The equation is y = 0.04485 + (0.89135–0.04485)/ (1 + x/0.29904)^1.07622^, with a correlation coefficient (R^2^) of 0.98295. **(B)** Standard linear curve of inhibition competitive ELISA. The linear equation was y = 0.6294x + 0.753, with a correlation coefficient (R^2^) of 0.8453.

A competitive inhibition ELISA method was performed to construct the standard curve and recovery test. The standard curve was constructed and the data obtained from the relationship between OTA concentration and its inhibition was analyzed using software Microcal Originpro 9.1. From the result shown in Figure 4A, typical calibration curve, the logistic equation was y = 0.04485 + (0.89135–0.04485)/(1 + x/0.29904)^1.07622^, with a correlation coefficient (R^2^) of 0.98295. The linear equation was y = 0.6294x + 0.753, with a correlation coefficient (R^2^) of 0.8453 (Figure 4B). In the present study, the half inhibitory concentration (IC_50_) was 0.2 ng/mL and the linear range to detect OTA was 0.06–0.6 ng/mL, which is defined as the concentration of OTA giving a 20–80% inhibition, and its lower detection limit (LOD) was 0.03 ng/mL, which was better, lower, than that reported in ELISA until now (Gyöngyösi-Horváth et al., 1996; Cho et al., 2005; Alarcón et al., 2006; Zhang et al., 2011, 2015; Pavón et al., 2012; Li et al., 2013; Venkataramana et al., 2015).

### Samples Detection Using mAb by ic-ELISA

Recovery test was carried out to evaluate the sensitivity of the established ic-ELISA assay. Non-contaminated corn was obtained, extracted, diluted, and spiked with OTA at different concentrations (20, 50, 100, and 150 ng/mL). The average recoveries within and between batches were from (84.21 ± 1.42) to (93.11 ± 1.83)%, with an average recovery of (89.315 ± 2.257)%, and the coefficient of variation (CV) ranged from 1.35 to 2.97% with an average CV of 2.182% (Supplementary Table 1). Real samples (corn flour, corn flakes, corn kernels, corn starch, and CH_2_OH) were randomly obtained from the local market for analysis. In addition, the matrix interference was evaluated by ic-ELISA at different concentrations of OTA mycotoxin. Artificially, OTA contaminated corn products were used for the assessment of the matrix effect, and the standard curves of ic-ELISA were developed in the PBS and the diluted matrix. For sensitivity of the ic-ELISA assay, the matrix effect was reduced from sample extract by 50-fold dilution, hence it was suitable for the determination of OTA in real samples. The collected samples were extracted and properly diluted for reducing the matrix effect, and to assess the accuracy of ic-ELISA for ochratoxins (OTA or OTB) detection using 6E5 mAb. The results showed that no ochratoxins (OTA and OTB) contamination was detected in these real corn samples (Supplementary Table 2).

### Construction and Analysis by Colloidal Gold Strip

**Figure 5:**
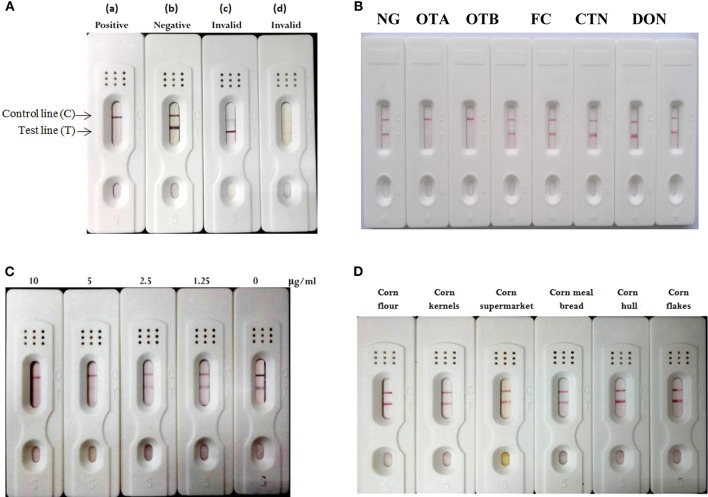
Construction and characterization of the colloidal gold strip test. **(A)** The explanation for strip test results. Positive is indicated if only stained line was in control line (a). Negative is indicated if both control and test zone were stained lines (b). An invalid result occurred because of no-formation of control line (c, d). **(B)** Cross-reactivity of the test strip with other toxins, such as OTB, CTN, FB1, DON, and ZEN. **(C)** Detection limit of colloidal gold strip test for OTA. **(D)** Detection of real samples for ochratoxins.

The gold-labeled mAb was used to construct colloidal gold strip for testing mycotoxins in samples (Ling et al., 2014; Liu et al., 2014; Wang et al., 2017). The model for describing the colloidal gold strip assay was shown in Figure 5A. OTA-BSA conjugate and HRP-conjugate were embedded onto nitrocellulose to form test line and control line, respectively. If the sample solution carried the sufficient ochratoxins (OTA or OTB), the reaction with the gold-labeled mAb will occur, so there is no gold-labeled mAb to bind to OTA-BSA conjugate embedded on test zone. This will not results in formation of red color appearing on the test line, and this is regarded as the positive result (a). In contrast, a formation of stained line (negative result) will be visible, indicating the absence of OTA or OTB toxin in the investigated sample (b). For accuracy of the procedure and operation, red line should always occur in the control zone regardless of the OTA or OTB presence or not. Conversely, if no red color appeared on the control line as shown in (c) and (d), it is defined as invalid results. Several mycotoxins other than OTA and OTB, such as CTN, FB1, DON, and ZEN were used to determine the cross-reactivity of the strip assay. If samples contain non-OTA or non-OTB toxin, formation of one red line occurs on the test line (Figure 5B), indicating that no cross-reactivity (high specificity) was observed. The result showed that colloidal gold strip test had high specificity to ochratoxins (OTA or OTB). For sensitivity assay, different concentrations (0–10 μg/mL) of OTA standard solution were used. The limit of detection was observed from Figure 5C, showing that the detection limit of this strip for OTA was 5 μg/mL. For examination of the robustness of this colloidal gold strip, real food matrices (corn flour, corn kernels, corn supermarket, corn meal bread, corn hull, and corn flakes) were collected and used in this evaluation, and the results in Figure 5D indicated that these samples were free of OTA and OTB contamination, and could be detected out within 5 min.

### Preparation and Analysis by Gold Nanoflowers (AuNFs)

**Figure 6:**
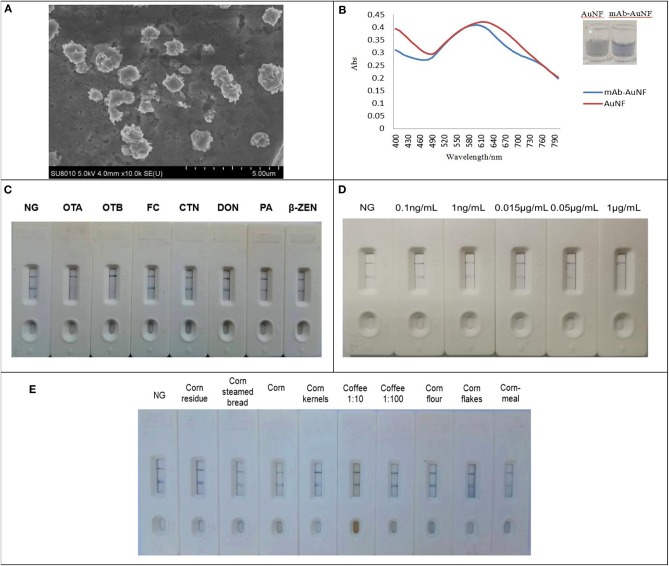
Construction and characterization of the gold nanoflowers strip test. **(A)** TEM image of AuNFs. **(B)** The UV-visible spectra of AuNFs. **(C)** Cross-reactivity of the test strip with other toxins. **(D)** Detection limit of AuNFs. **(E)** Detection of real sample solution for ochratoxins.

Properly prepared AuNFs were characterized to show its shape by TEM image as described in Figure 6A. For gold nanoflowers (AuNFs) preparation, successful progress of AuNFs was monitored as shown in Figure 6B by UV-vis spectra at a wavelength range of 400–800 nm, and the absorption wavelength up to 605 nm was selected as the maximum peak for anti-OTA mAb-AuNFs and AuNFs (Thobhani et al., 2010). The UV-vis spectra in this figure demonstrated that AuNFs solution was successfully prepared and AuNFs was conjugated successfully with mAb. For determining the optimal pH of AuNFs solution for AuNFs-mAb, 0.1 mol/L K_2_CO_3_ was used to adjust the solution, and the final optimal pH of 9.0 was determined to be suitable for the reaction between mAb and AuNFs (Supplementary Figure 1). Furthermore, the mAb was dropped to the AuNFs solution with final optimal pH of 9.0. In this study, 3 μL of AuNFs-mAb was regarded as optimum protein binding volume (Supplementary Figure 2). The principle of the AuNFs strip was the same to Figure 5A. The specificity of the strip assay was analyzed by different toxins including OTA, OTB, CTN, FB1, DON, and ZEN. Result in Figure 6C showed that high specificity to OTA was observed among these tested toxins with exception of cross-reactivity to OTB (Supplementary Table 3). To determine the limits of strip assay, different concentrations of OTA standard solution were subjected to the AuNFs strip test. As shown in Figure 6D, the red color disappeared at 1 μg/mL, indicating that the limit of detection for ochratoxins (OTA or OTB) by this nanoflowers strip was 1 μg/mL (Supplementary Figure 3). AuNFs strip test established in our work were applied to test ochratoxins (OTA or OTB) in real samples, such as corn, corn kernels, corn meal, corn flour, corn residue, and coffee. The results from Figure 6E showed that two red bands appeared on the test strips, and all the reaction could be finished within 5 min. The results mean that these samples were free of OTA and OTB contamination (Supplementary Table 4). Therefore, the use of multi-branched AuNF as probe can be successfully deployed in the detection of ochratoxins (OTA or OTB) in real samples.

## Conclusion

A sensitive and specific anti-ochratoxins (OTA and OTB) mAb was generated from the 6E5 stable hybridoma cell line. This antibody with 3.7 × 10^8^ L/mol high affinity was successfully used to establish an ic-ELISA, colloidal gold strip and nanoflowers gold strip immunoassay for detection of ochratoxins (OTA and OTB). The linear range of optimized ic-ELISA was 0.06–0.6 ng/mL with IC50 of 0.2 ng/mL. The lower limit of detection (LOD) of ic-ELISA, colloidal and nanoflowers gold strip were 0.03 ng/mL, 5 and 1 μg/mL, respectively. Therefore, the mAb described in this study is suitable, sensitive and specific for use in these three established immunoassays for ochratoxins (OTA and OTB) detection in real samples. Further work focusing on increased sensitivity of these methods of ochratoxins detection in real samples is recommended.

## Data Availability Statement

All datasets generated for this study are included in the article/Supplementary Material.

## Ethics Statement

The animal study was reviewed and approved by the Animal Experiment Committee of Fujian Agriculture and Forestry University. This study was performed according to principles of laboratory animal care were followed and all procedures were conducted according to the guidelines established by the National Institutes of Health, and every effort was made to minimize suffering.

## Author Contributions

RW and SW designed the experiments and wrote the manuscript. MF, SL, RW, and XL performed all the experiments. JY, SX, KW, ST, and HE performed a few experiments and data analysis. All authors read and approved the final manuscript.

### Conflict of Interest

The authors declare that the research was conducted in the absence of any commercial or financial relationships that could be construed as a potential conflict of interest.
