# Supplementary material for: Development of ELISA and Lateral Flow Immunoassays for Ochratoxins (OTA and OTB) Detection Based on Monoclonal Antibody
Source: Front Cell Infect Microbiol. 2020 Mar 6;10:80. doi: 10.3389/fcimb.2020.00080 (PMC7067699; doi:10.3389/fcimb.2020.00080)

**Development of ELISA and lateral flow immunoassays for ochratoxin A (OTA) detection based on monoclonal antibody**

Mohamed Fadlalla^a,c^, Sumei Ling^a,b,c^, Xiulan Li^a,c^, Jun Yuan^a,c^, Shiwei Xiao^a,b^, Ke Wang^a,c^, Shuqin Tang^a,c^, Hoyda Elsir^a,c^, Shihua Wang^a,b,c∗^

^a^ *Fujian Key Laboratory of Pathogenic Fungi and Mycotoxins, Fujian Agriculture and Forestry University, Fuzhou 350002, China*

^b^*Key Laboratory of Biopesticide and Chemical Biology of the Education Ministry, Fujian Agriculture and Forestry University, Fuzhou 350002, China*

^c^ *School of Life Sciences, Fujian Agriculture and Forestry University, Fuzhou 350002, China*

* Correspondence: wshyyl@sina.com. Tel. /Fax: +85-591-8798-4471.

Appendix A. Supporting information

Supplementary 1

Preparation of colloidal gold nanoparticles (CGNs)

Glassware and other tools used in this process were kept sterile as any particles on the equipment or solutions used could destabilize the gold. Colloidal gold was prepared by using a seed growth strategy using trisodium citrate as reducing agent. In preparation of 0.01% of chloroauric acid solution, 1mL 1% (m/v) HAuCl4 solution was added to conical flask containing 100 mL of deionized water then boiled until multiple bubbles were observed. After that, 2 mL of 1% (w/v) trisodium citrate solution was added and the solution was allowed to boil for 6 minutes with constant stirring. The color of the solution changed to wine-red after 3 min from citrate addition was added. The flask was then removed from heater and placed at room temperature. After the solution was cooled, deionized water was added to make up the solution to 100 mL. The colloidal gold solution was stored at 4 °C.

Transmission electron microscopy observation

Appropriate amount of the nanoflower solution were taken, place it on the Formvar film copper net, absorb excess liquid, dry it in an oven, and observe whether the size and dispersion degree of the nano flower particles are uniform under electron microscope (40000 times) and calculate the average diameter of the nanoflower particles.

Supplementary Table1

**Table 1.** The recovery and coefficients of variation in real sample with spiked OTA


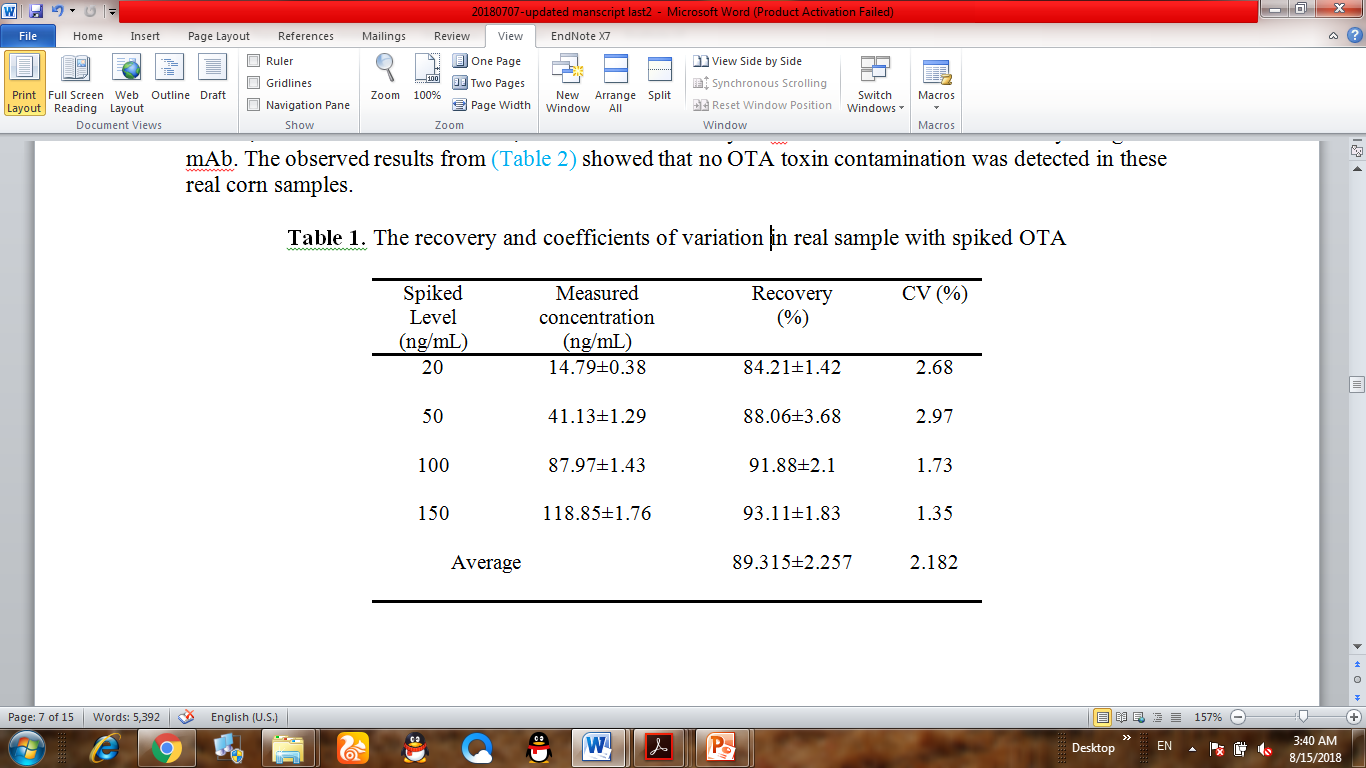


± shows the average deviation from the mean. Data were as the average value. The coefficient variation (CV) was described as the ratio of the standard deviation to the average in the recovery test.

Supplementary Table 2

**Table 2.** Analysis of OTA toxin in real sample.


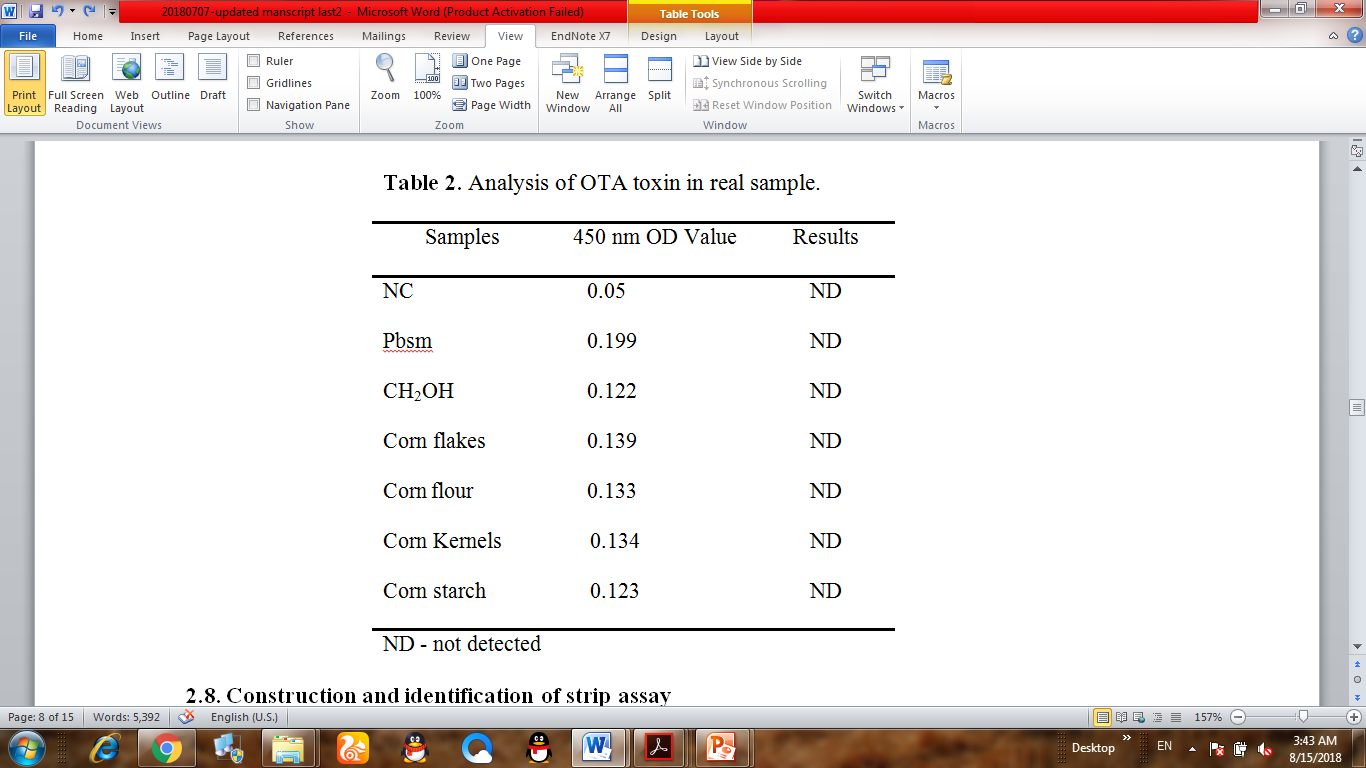


ND means no OTA was detected in samples tests.
PBSM = means 5% nonfat dry milk/PBS

Supplementary Fig.1


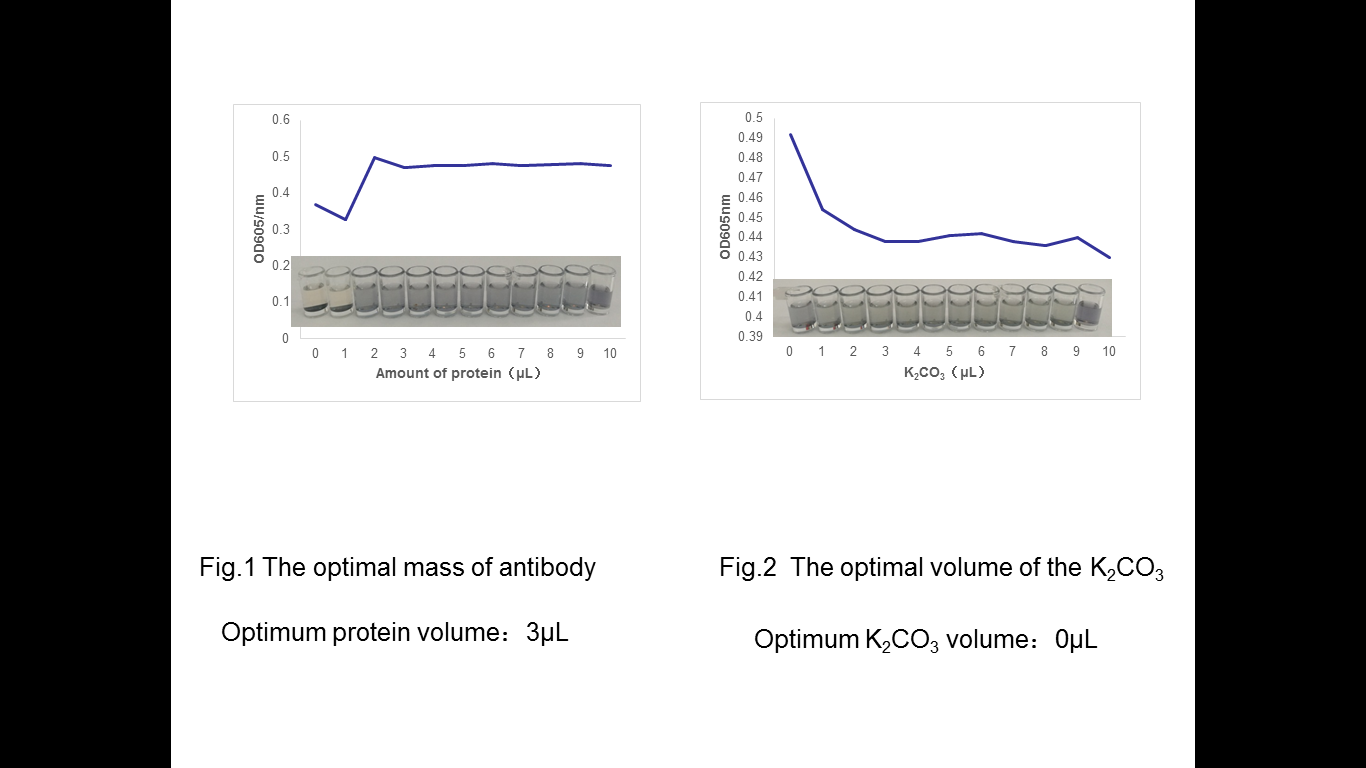


Fig.1. The optimal volume of the K2CO3

Supplementary Fig.2


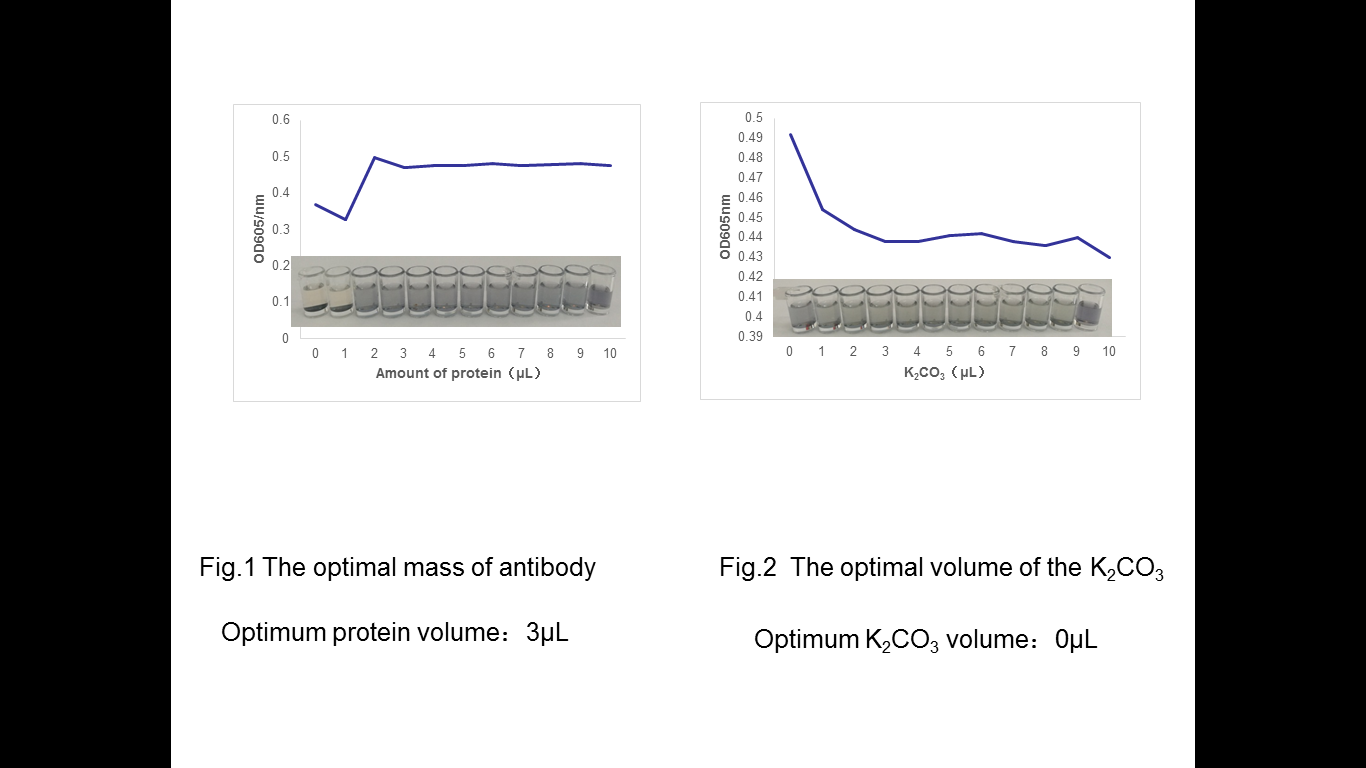


Fig.2. The Nanoflowers optimal mass of antibody

Supplementary table 3

Table 3. Nanoflowers specificity and cross-reactivity


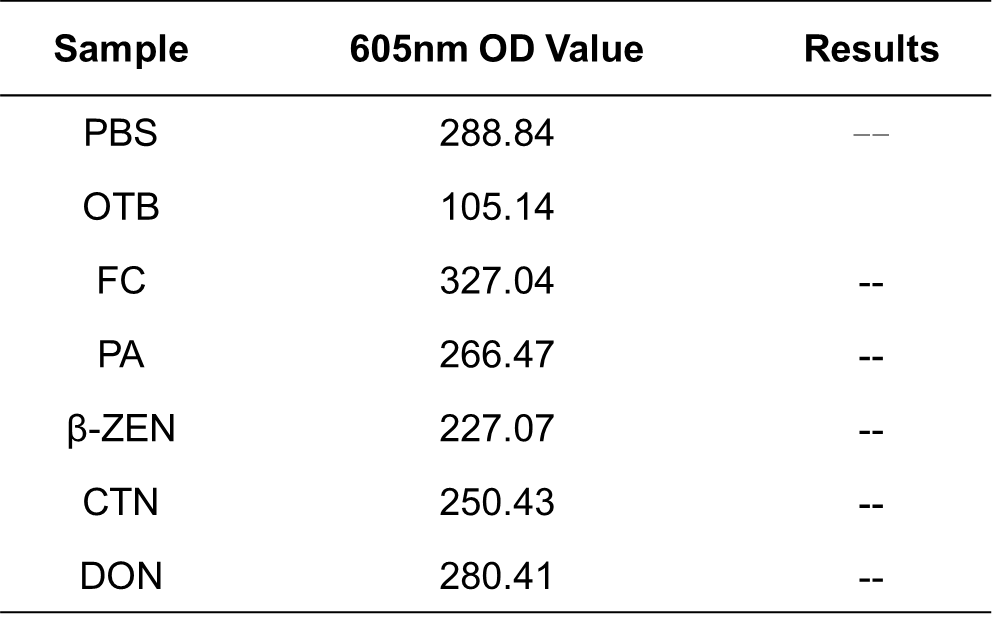


Supplementary Fig. 3.


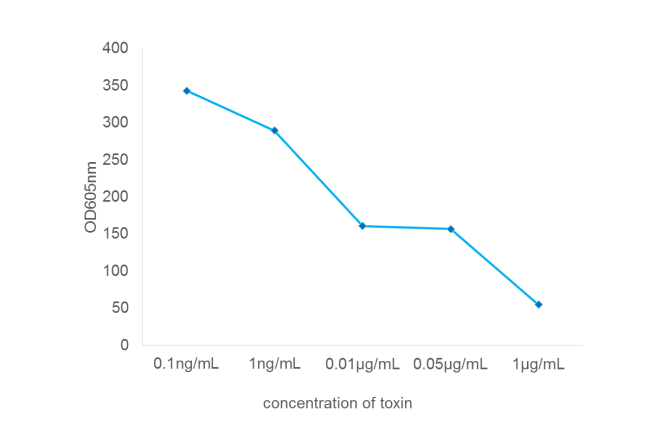

Fig. 3 Nanoflowers sensitivity test of the strip

Supplementary table 4

Table 4. Detection of real sample solutions for OTA.


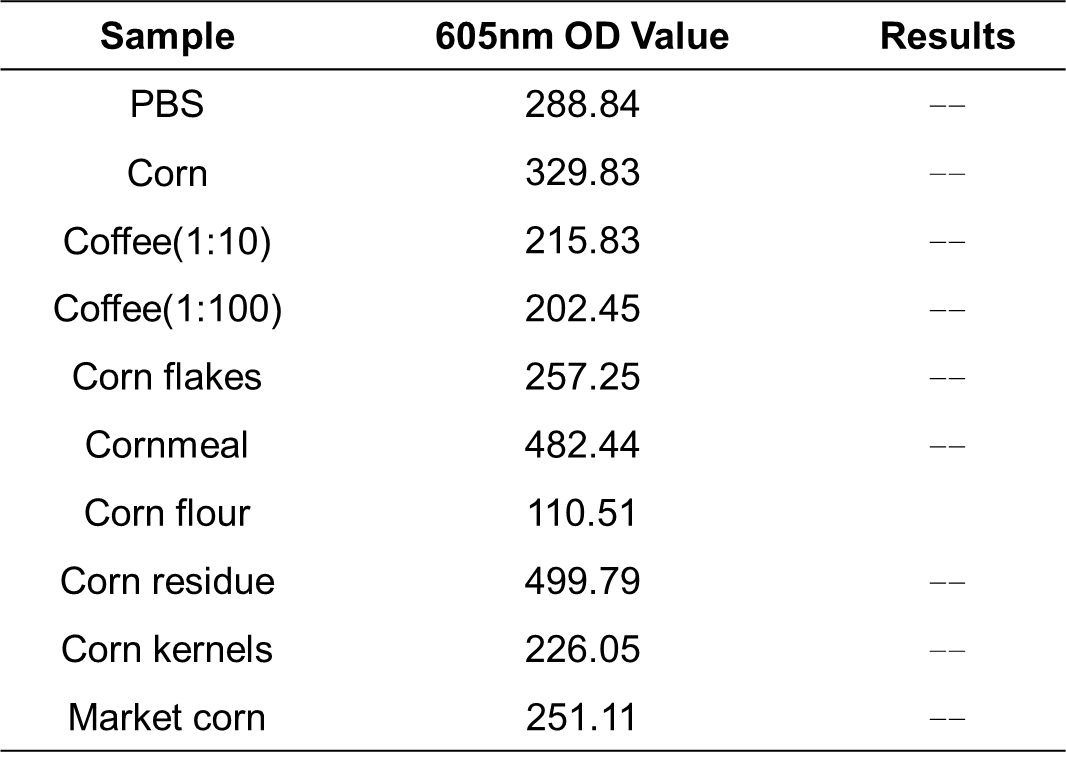

Supplement: Supplementary file 1 [file Data_Sheet_1.docx]
